# Functional response of wolves to human development across boreal North America

**DOI:** 10.1002/ece3.5600

**Published:** 2019-08-30

**Authors:** Tyler B. Muhly, Cheryl A. Johnson, Mark Hebblewhite, Eric W. Neilson, Daniel Fortin, John M. Fryxell, Andrew David M. Latham, Maria C. Latham, Philip D. McLoughlin, Evelyn Merrill, Paul C. Paquet, Brent R. Patterson, Fiona Schmiegelow, Fiona Scurrah, Marco Musiani

**Affiliations:** ^1^ Forest Analysis and Inventory Branch Ministry of Forests, Lands, Natural Resource Operations and Rural Development Government of British Columbia Victoria BC Canada; ^2^ Science and Technology Branch of Environment and Climate Change Canada Ottawa ON Canada; ^3^ Wildlife Biology Program Department of Ecosystem and Conservation Sciences W.A. Franke College of Forestry and Conservation University of Montana Missoula MT USA; ^4^ Department of Biological Sciences University of Alberta Edmonton AB Canada; ^5^ Department of Biology Centre d'étude de la forêt Université Laval Québec QC Canada; ^6^ Department of Integrated Biology University of Guelph Guelph ON Canada; ^7^ Manaaki Whenua Landcare Research Lincoln New Zealand; ^8^ Department of Biology University of Saskatchewan Saskatoon SK Canada; ^9^ Department of Geography University of Victoria Victoria BC Canada; ^10^ Wildlife Research and Monitoring Section Ministry of Natural Resources and Forestry Trent University Peterborough ON Canada; ^11^ Department of Renewable Resources University of Alberta c/o Yukon Research Centre Whitehorse YT Canada; ^12^ Transmission Line and Civil Construction Manitoba Hydro Winnipeg MB Canada; ^13^ Department of Biological Sciences Faculty of Science University of Calgary Calgary AB Canada

**Keywords:** boreal forest, ecosystem conservation, forestry, functional response, habitat selection, roads, trade‐offs, wolves

## Abstract

**Aim:**

The influence of humans on large carnivores, including wolves, is a worldwide conservation concern. In addition, human‐caused changes in carnivore density and distribution might have impacts on prey and, indirectly, on vegetation. We therefore tested wolf responses to infrastructure related to natural resource development (i.e., human footprint).

**Location:**

Our study provides one of the most extensive assessments of how predators like wolves select habitat in response to various degrees of footprint across boreal ecosystems encompassing over a million square kilometers of Canada.

**Methods:**

We deployed GPS‐collars on 172 wolves, monitored movements and used a generalized functional response (GFR) model of resource selection. A functional response in habitat selection occurs when selection varies as a function of the availability of that habitat. GFRs can clarify how human‐induced habitat changes are influencing wildlife across large, diverse landscapes.

**Results:**

Wolves displayed a functional response to footprint. Wolves were more likely to select forest harvest cutblocks in regions with higher cutblock density (i.e., a positive functional response to high‐quality habitats for ungulate prey) and to select for higher road density in regions where road density was high (i.e., a positive functional response to human‐created travel routes). Wolves were more likely to use cutblocks in habitats with low road densities, and more likely to use roads in habitats with low cutblock densities, except in winter when wolves were more likely to use roads regardless of cutblock density.

**Main conclusions:**

These interactions suggest that wolves trade‐off among human‐impacted habitats, and adaptively switch from using roads to facilitate movement (while also risking encounters with humans), to using cutblocks that may have higher ungulate densities. We recommend that conservation managers consider the contextual and interacting effects of footprints when assessing impacts on carnivores. These effects likely have indirect impacts on ecosystems too, including on prey species.

## Graphical abstract

We deployed GPS‐collars on 172 wolves and evaluated resource selection across boreal ecosystems encompassing over a million square kilometers of North America. Wolves traded‐off among human impacted habitats, and more specifically adaptively switch from using roads to facilitate movement (while also risking encounters with humans), to using cutblocks that may have higher ungulate densities. We therefore recommend that conservation managers consider the contextual and interacting effects of human footprints when assessing impacts on carnivores and on their prey.

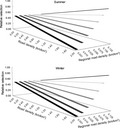

## 1 INTRODUCTION

**Figure 1:**
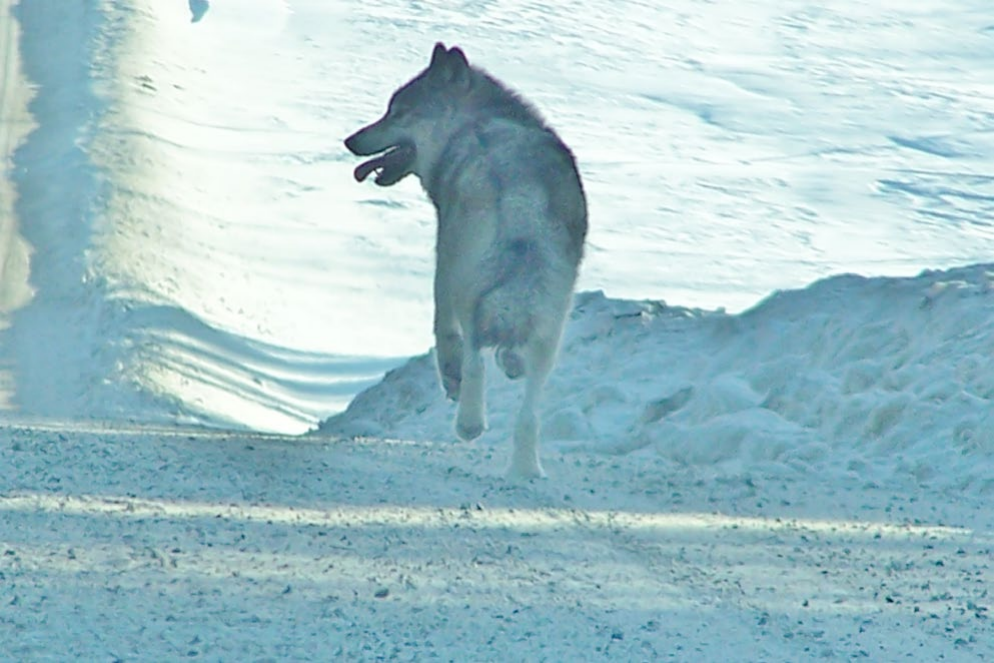
A wolf (*Canis lupus*) in the Boreal Forest of Northern Alberta, Canada, where roads were presumably more energetically favorable for travel (i.e., lower sinking depths)

The influence of humans on large carnivore populations is a worldwide conservation concern. The number of terrestrial mammalian carnivores threatened by exposure to roads in particular is increasing across the globe, with Asia and North America being hotspots for species at risk (Ceia‐Hasse, Borda‐de‐Agua, Grilo & Pereira, 2017). Humans can also have indirect effects on ecosystems by influencing carnivore distribution and abundance (also including wolves', *Canis lupus*, Figure 1), which in turn can induce changes in prey distribution, herbivory, and vegetation (Ripple et al., 2014). In North America, wolves are important predators of mammalian herbivores, from large ungulates such as moose (*Alces alces*) to medium sized animals such as beaver (*Castor canadensis*). In addition, wolves may influence the abundance of prey species via predator‐mediated apparent competition (Holt, 1977; Serrouya, McLellan, van Oort, Mowat, & Boutin, 2017) and these interactions may also be affected by human disturbance.

Wolf predation is often influenced by infrastructure related to natural resource development (i.e., footprint), with varying effects on prey. For example, rapid energy and forestry development in the boreal forest of North America (Timoney & Lee, 2001; Venier et al., 2014) create movement corridors for predators, particularly wolves, therefore enhancing predation (e.g., Demars & Boutin, 2017; Dickie, Serrouya, Scott McNay, & Boutin, 2017; Latham, Latham, Boyce, & Boutin, 2011; Paquet, Alexander, Donelon, & Callaghan, 2010; Whittington et al., 2011). In addition, forestry may produce early seral forests that support higher abundance of prey such as moose and deer (*Odocoileus* spp.), and in turn higher abundance of predators such as wolves (e.g., Dussault, Courtois, & Ouellet, 2006; Houle, Fortin, Dussault, Courtois, & Ouellet, 2010; Peters, Hebblewhite, DeCesare, Cagnacci, & Musiani, 2012). Human footprint can also degrade habitat by increasing wolf‐caused mortality on sensitive species such as woodland caribou (*Rangifer tarandus*), which are listed as *Threatened* under the Species at Risk Act in Canada (Fortin et al., 2017; Hervieux et al., 2013; Wittmer, Sinclair, & McLellan, 2005). This has led to the implementation of intensive wolf management in some areas of the boreal forest (Hervieux, Hebblewhite, Stepnisky, Bacon, & Boutin, 2014). Predicting how top carnivores, including wolves, respond to human‐induced changes to habitat at scales that are commensurate with the wide‐ranging land use changes underway in the boreal forest is therefore necessary for carnivore conservation, ecosystem management, and threatened species recovery.

A functional response in habitat selection, including human footprint, occurs when habitat selection varies as a function of the availability of that habitat. Such functional responses are likely common where animals make trade‐offs, for example, between mortality risk from humans and food (Mysterud & Ims, 1998). Functional responses have been revealed in a variety of mammals, including polar bears (*Ursus maritimus*; Mauritzen et al., 2003), raccoons (*Procyon lotor*; Tardy, Massé, Pelletier, Mainguy, & Fortin, 2014), and moose (Beyer, Ung, Murray, & Fortin, 2013; Street et al., 2015). Statistical models of wildlife habitat selection that include functional responses have recently been developed (Gillies et al., 2006; Matthiopoulos, Hebblewhite, Aarts, & Fieberg, 2011; Moreau, Fortin, Couturier, & Duchesne, 2012) to assess how wildlife make trade‐offs in habitat selection as habitat availability changes.

Previous studies show that wolves exhibit a highly variable response to human footprint. Wolves may avoid human footprint (Benson, Mahoney, & Patterson, 2015; Mladenoff & Sickley, 1998; Oakleaf et al., 2006) or select for it (Bowman, Ray, Magoun, Johnson, & Dawson, 2010; Lesmerises, Dussault, & St‐Laurent, 2012; Paquet et al., 2010; Whittington, St. Clair, & Mercer, 2005), and some researchers concluded wolves were indifferent to human activity (e.g., Mech, Fritts, Radde, & Paul, 1988). However, there is potential for functional responses in wolves, as most assessments of resource selection occurred in areas where habitat availability did not vary, and thus the results of these studies represented resource selection within a unique habitat availability condition. The plasticity in wolf response to human footprint has recently been suggested as potentially indicating functional responses (e.g., Hebblewhite & Merrill, 2008; Houle et al., 2010; Newtonet al., 2017). However, studies to date modeled wolf response to human footprint across limited spatial scales (i.e., resource availability defined over 100's to 1,000's of square kilometers). Functional responses to human footprint across 1,000,000's of km^2^ using data from a large sample of individuals, like in this study, are required to capture and understand the full range of responses to environmental conditions experienced by wide‐ranging species such as wolves.

In Canada's boreal forest, forestry operations produce areas of partially or completely removed and disturbed vegetation, which are referred to as “cutblocks” (Grindal & Brigham, 1999). Wolves might select for cutblocks, as these areas are characterized by early seral vegetation and abundance of primary prey species (Bowman et al., 2010; Kittle et al., 2017; Peters et al., 2012). In the boreal forest, wolves might also select for roads, as these provide increased travel efficiency and ease of finding prey (Dickie et al., 2017; Newton et al., 2017; Paquet et al., 2010; Whittington et al., 2011). However, wolf selection for roads might be diminished by perceived increased risk of human‐wolf interactions there, with potential trade‐off between ease of travel and fear of encounters with humans (Benson et al., 2015; Lovari, Sforzi, Scala, & Fico, 2007). Finally, wolves' selection of forestry cutblocks and roads may also interplay. For example, Kittle et al. (2017) suggested that wolves are more likely to use linear features, including roads, that facilitate movement when prey abundance is low, but may switch to using cutblocks to find prey in landscapes with higher prey densities.

We empirically modeled wolf selection of human footprint across 1,000,000 km^2^ of the boreal forest of Canada. Specifically, we examined two aspects of human activity (forestry cutblocks and roads) that may act as opposing resources for wolves. We predicted that wolves would increase use of forest harvest cutblocks as their availability increases. We tested how wolves responded to roads, knowing that roads could be selected for ease of travel, but avoided for fear of humans there. We also tested for interactions of cutblock and road availabilities in wolf habitat selection. Finally, we evaluated model generalizability at predicting wolf distribution outside of conditions under which the model was trained. Our study therefore provides one of the most spatially extensive and large sample (*n* = 172 wolves) tests of how predators respond to human footprint.

## 2 METHODS

### 2.1 Study area and wolf data

**Figure 2:**
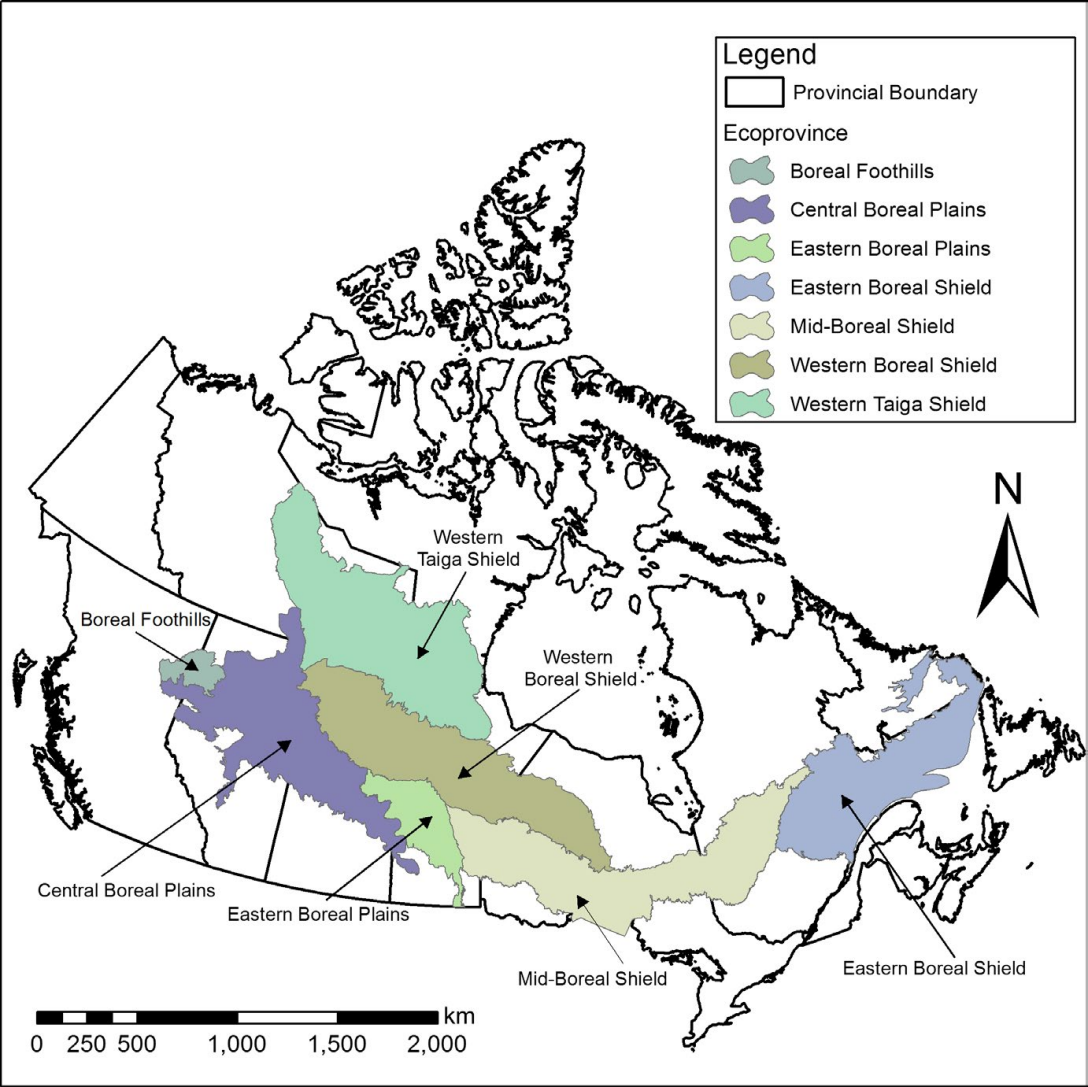
Boreal forest of Canada and its seven ecoprovinces, where wolf location data was available

The study occurred in the boreal forest of North America, which spans >1,000,000 km^2^ from Labrador to the Yukon across central Canada (Figure 2). The study area consisted of seven ecoprovinces (from west to east): boreal foothills, central boreal plains, western taiga shield, western boreal shield, eastern boreal plains, midboreal shield, and eastern boreal shield. Ecoprovinces are areas of uniform climate, geological history, and physiography (Demarchi, 1996). See Brandt (2009) for a detailed review of the ecology of the boreal forest and its ecoprovinces.

First, we compiled Global Positioning System (GPS) telemetry datasets from 255 wolves collected by research groups and government agencies from across Canada from 1997 to 2013 (see Table S1). The fix rate interval of the wolf location data was subsampled to either be weekly (data from Ontario, Canada) or daily (all other data). Location data were collected by an assortment of GPS telemetry collar makes and models. We removed from the dataset any wolves with fewer than 40 telemetry locations collected, therefore achieving a number adequate to determine the distribution of wolf packs in a region (Fuller & Snow, 1988). Results by Fuller and Snow (1988) indicated that territories described from >40 locations should be large enough (85%–90% of total size obtained with more locations) to determine whether another wolf pack (potentially “competing” for habitat selection) might reside between two territories. We also removed wolves with yearly home ranges overlapping with each other's (indicating same pack) and kept the individual with more locations or with home ranges not overlapping with the edge of the study area. This resulted in further reduction of the dataset as many participating studies had collared multiple individuals in packs (often to allow continued monitoring of packs also after a given accidental collar failure). However, with this strategy we avoided issues of pseudo‐replication, which could have occurred as wolf packs compromise individuals whose movements (and therefore habitat selection) are not fully independent (see Benson & Patterson, 2015). After filtering for these criteria, 172 individual wolves remained in the dataset. Sex and age distribution were as follows: 83 females, 77 males and 11 of unknown or unreported sex, and 140 adults and 32 classified as yearlings or pups. In total, 83% of wolf location data used in the analysis were collected between 2006 and 2012, and <10% were collected prior to 2005. Capture and handling of collared wolves followed approved animal care protocols (see Table S1).

### 2.2 Resources measured at used and available locations

Our model evaluated individual wolf selection of resources within an area they could occupy over the course of a year based on their movement ecology. We sampled resources (i.e., habitat) available to each individual wolf at locations up to a maximum distance of one wolf home range away from each wolves' telemetry (i.e., used) locations. Thus, our sample of available resources was representative of an area that could realistically be accessed by each individual wolf we monitored. We acknowledge that some of these areas may not have been easily accessible because of the territorial nature of neighboring wolf packs, and territoriality of wolves could limit the inference to be gained by our study with regard to habitat used versus available. In addition, in this study creation of available points was restricted to the ecoprovince where the GPS locations of a given wolf occurred, as the human footprint data was available by ecoprovince. However, wolves could consider as available some areas outside of their ecoprovince too. Overall, our sample of 172 GPS collared wolves guaranteed that the areas frequented, and the areas around, which were potentially reachable by each wolf were highly variable with regard to environmental conditions. Therefore, our available sample adequately represented the variability of habitat within and near each individual wolf's range likely without biases, regardless of the presence or absence of neighboring wolves.

We estimated the distance across a wolf home range from our data by measuring the maximum net displacement (i.e., Euclidean distance) between all telemetry locations for each individual wolf using the “ltraj” function from the “adehabitat” library (Calenge, 2006) in program R (R Core Team, 2013). Telemetry locations were collected over an approximately 1‐year period for each wolf and thus the maximum net displacement reasonably represented the distance across an annual home range of a wolf. Overall, we created a frequency distribution of maximum net displacements for the 172 wolves, except we removed the top 5% displacement values as outliers (accounting for potential extraterritorial forays, Messier, 1985). For each telemetry location (“used” by wolves), we sampled 10 “available” locations at random directions and distances drawn randomly from the frequency distribution. These available locations were specific to the individual wolf from which the sample location was drawn (see Figure S1).

Thus, the spatial distribution of habitat available to an individual wolf was defined based on a biological parameter: The maximum net displacement by each radio collared wolf over the course of a year. In addition, available habitat was further constrained in our study to an area that is reasonably and practically accessible to an individual wolf over the course of a year, based on quantified movement capabilities (van Moorter et al., 2013), rather than across the entire distribution of the wolf population in the boreal forest–i.e., a vast region, not practically available to each wolf.

Our methodology shared similarities with an approach based on second‐order selection, specifically because we consider habitat information outside of home ranges (Boyce, 2006). However, we defined the domain of availability as the area both inside and outside (according to movement capacities) of home ranges. Our study thus includes elements of both second‐order selection (i.e., outside home ranges) and third‐order selection (i.e., within home ranges), as in Boyce et al. (2003), Gagné, Mainguy, and Fortin (2016) and Losier et al. (2015).

Habitat selection is a multiscale process (Boyce, 2006), and studies can assess habitat selection at multiple scales (see McGarigal, Wan, Zeller, Timm, & Cushman, 2016). Even movement analysis such as step selection functions (Fortin et al., 2005) is generally based on habitat information taken not only within but also outside of home ranges (i.e., a number of random steps should fall outside of home ranges). Boyce et al. (2003) showed how habitat selection can be studied at multiple scales based on the same observed locations, but on random locations distributed over different domains of availability. Some of the scales Boyce et al. (2003) considered were based on a domain of availability that exceeded the home ranges of individuals, an approach similar to ours. As with any habitat selection model, the interpretation of our model should be done while considering the spatial domain of availability. Specifically, our study evaluates habitat features that are used more or less than expected given the availability of those features within an area that is reasonably and practically accessible to an individual wolf over the course of a year.

Changes in forest landscapes are temporally dynamic. However, environmental data sets comprehensively covering the telemetry period (ideally at regular time intervals) and the whole study area were not available. We had to rely on datasets that were diligently assembled, largely by Environment Canada. A standardized methodology was developed and implemented by Pasher, Seed, and Duffe (2013) to create a single geospatial dataset representing anthropogenic disturbances across a significant portion of Canada's boreal ecosystem. The boreal ecosystem anthropogenic disturbances data are a vector disturbance dataset of individual linear and polygonal disturbance types that were manually collected through the interpretation of 2008–2010 Landsat imagery at a 1:50,000 viewing scale. For our study, we compiled spatial datasets of forest cutblock density, road density and vegetation biomass (i.e., a proxy of wolf prey) to estimate habitat at locations used by and available to wolves. We relied on Environment Canada's disturbance maps, which were created using Landsat imagery, to estimate (a) forest cutblock density (km^2^/km^2^) and (b) road density (km/km^2^), both at a 1 km^2^ spatial resolution. These spatial layers represented the most recent and comprehensive attempt available at assessing human footprint in the study area (Pasher et al., 2013), similar to the satellite imagery data described below.

Wolves prey and rely on a variety of ungulate species across boreal North America, including moose, woodland caribou, and deer (Latham et al., 2011; Latham, Latham, Knopff, Hebblewhite, & Boutin, 2013; Messier, 1994). However, data on wolf prey densities were unavailable. We therefore used the average summer NDVI value (i.e., peak of vegetation productivity in the boreal forest) as an indicator of prey biomass distribution throughout that particular year, which is known to correlate with high‐quality forage (e.g., Pettorelli et al., 2011; Street et al., 2015). NDVI information was obtained from MODIS data collected by the U.S. Geological Survey Earth Resources Observation and Science Center at 16‐day intervals during the summer (June 1 to September 30) at a 1 km^2^ spatial resolution.

### 2.3 Resource selection function analysis

We used a resource selection function (RSF) approach (Boyce & McDonald, 1999; Johnson, Nielsen, Merrill, McDonald, & Boyce, 2006; Manly, McDonald, Thomas, McDonald, & Erickson, 2007) to model wolf occurrence across boreal Canada as a function of density of forest harvest cutblocks, density of roads, and vegetation biomass estimated using the normalized difference vegetation index (NDVI). We estimated the values of these covariates at wolf locations using point sampling tools in ArcGIS 10.1. Resources measured at locations used by wolves were compared with those measured at locations available to wolves in a binomial mixed‐effects regression model (Gillies et al., 2006; Hebblewhite & Merrill, 2008). The effect of habitat availability on resource selection was modeled with interaction terms in a GFR (Matthiopoulos et al., 2011) to test for functional responses of wolves to human footprint. GFRs extend the RSF approach to enable it to estimate generalized functional responses from spatial data. GFRs employ data from several sampling instances characterized by diverse profiles of habitat availability. In this study, we measured interaction terms of average forestry cutblock density and road density at locations available to wolves, by ecoprovince (see above).

We developed seasonal models of wolf resource selection, including summer (June 1 to September 30) and winter (October 1 to May 31). We tested for collinearity of habitat covariates using a Pearson correlation and found none that were highly correlated (|*r*| > .7; sensu Boyce, Vernier, Nielsen, & Schmiegelow, 2002). In addition, we calculated variance inflation factors (VIFs) to remove covariates in case they had a VIF >10 (high collinearity, Neter, Wasserman, & Kutner, 1990), and none was found.

In regression analyses, we employed generalized linear mixed models (GLMMs) in the package “lme4” (Bates, Maechler, Bolker, & Walker, 2015) in R 3.0.2 (R Core Team, 2013) with random intercepts for individual wolves. Models were fit with random slopes for individual wolves for each fixed‐effects covariate (i.e., NDVI, cutblock density and road density). Random intercepts and slopes were included to account for unbalanced sample sizes among wolves and for individual variability in wolf selection of resources, when estimating fixed effects of the sampled population. To estimate functional responses to cutblocks and roads, we also included the following covariates as interaction terms: (a) average cutblock density and (b) average road density, in each ecoprovince (sampled from locations available to wolves, see above). Models were fit with the bobyqa optimizer (R Core Team, 2013).

### 2.4 Mapping wolf resource selection, model generalizability and validation

We constructed maps of wolf resource selection across boreal Canada at a 9 km^2^ spatial resolution using the fixed‐effect coefficients from the GFR model for each season. We also tested for model generalizability (i.e., the ability of the model to predict wolf distribution as accurately with new data as with the model training data; Vaughan & Ormerod, 2005) using a k‐fold validation approach.

Using groups of withheld data rather than independent data to test generalizability makes it difficult to distinguish between errors in (a) overfitting (i.e., modeled idiosyncrasies in the data) and (b) transportability (i.e., inability of the model to predict the species–environment relationships outside of conditions under which the model was trained; Vaughan & Ormerod, 2005). However, overfitting is typically tested by bootstrapping from the training dataset (Vaughan & Ormerod, 2005). The k‐fold validation approach we used (a form of bootstrapping) can also test for overfitting. In addition, our k‐fold validation was designed to subdivide the data spatially (i.e., in ecoprovinces). Thus, it explicitly tested for generalizability of models built from wolf location data outside an ecoprovince on wolf location data within an ecoprovince. Our approach was the only reasonable alternative, given the lack of a broad‐scale independent dataset (e.g., from wolves in a similarly large expanse of boreal forest).

We evaluated model generalizability by comparing used versus expected numbers of wolf locations in each relative RSF probability bin for each withheld ecoprovince (sensu Boyce et al., 2002). We considered models that predicted the frequency of used locations within RSF bins as having good generalizability, for example, the frequency of predicted and used locations had strong goodness‐of‐fit statistics, including, ideally, high *R* ^2^ values, slopes approaching 1 and intercepts 0.

## 3 RESULTS

### 3.1 Wolves' functional responses to infrastructure

We obtained wolf locations (*n* = 604,650) from 172 GPS collared wolves from seven ecoprovinces across boreal Canada (Figure 2). We sampled a range of 5–33 wolves in each ecoprovince in the summer and 2–44 wolves in each ecoprovince in the winter (Table 1). Average road density by ecoprovince (i.e., average value of road density sampled at available locations in the ecoprovince) ranged from 0.001 km/km^2^ in the western taiga shield to 0.122 km/km^2^ in the boreal foothills (Table 1). Ecoprovince cutblock density ranged from 0 km^2^/km^2^ in the western taiga shield to 0.122 km^2^/km^2^ in the boreal foothills (Table 1).

**Table 1:** Number of wolves monitored with GPS‐telemetry in summer and winter, and average road and cutblock densities in sampled areas available to wolves in seven ecoprovinces of boreal Canada

Ecoprovince name	Number of wolves		Average road density (km/km^2^)		Average cutblock density (km^2^/km^2^)	
	Summer	Winter	Summer	Winter	Summer	Winter
Western taiga shield	6	14	0.001	0.001	0.000	0.000
Central boreal plains	33	39	0.085	0.084	0.041	0.041
Western boreal shield	26	44	0.027	0.027	0.009	0.010
Boreal foothills	26	2	0.122	0.011	0.122	0.010
Eastern boreal plains	5	12	0.078	0.074	0.011	0.013
Eastern boreal shield	8	9	0.017	0.017	0.115	0.116
Midboreal shield	13	33	0.044	0.048	0.062	0.078

**Figure 3:**
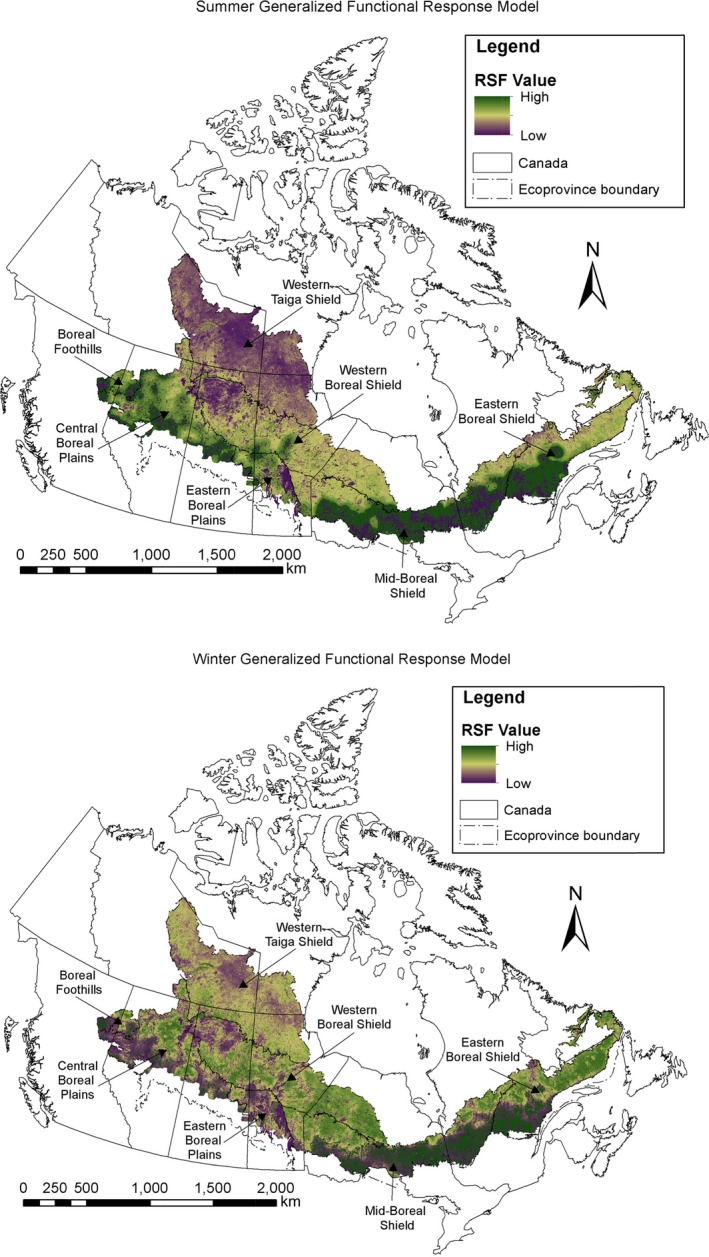
Wolf habitat selection across boreal Canada in summer (top) and winter (bottom) estimated using a generalized functional response (GFR) model. Resource selection function (RSF) values are displayed using histogram equalization, i.e., each range contains approximately the same number of pixels

**Figure 4:**
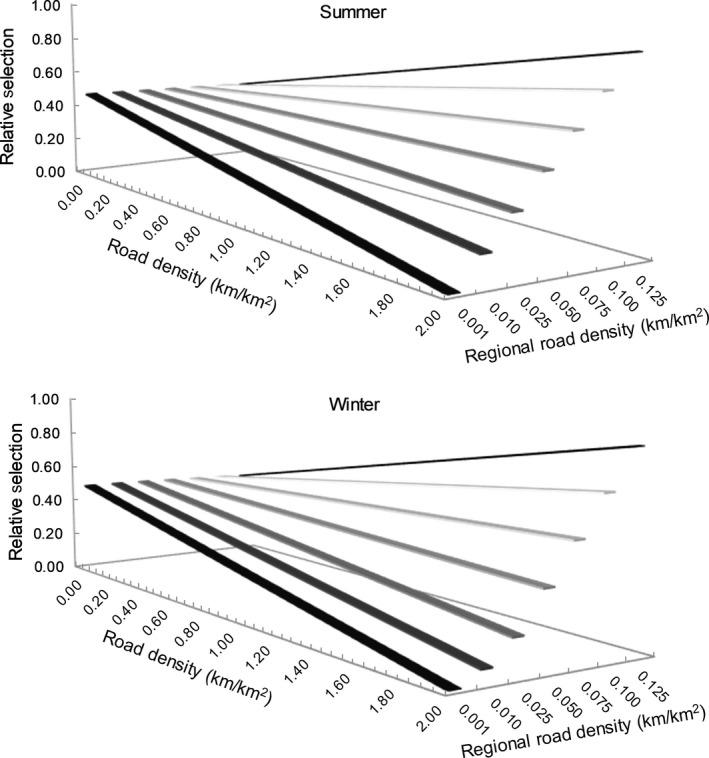
Relative selection by wolves of road density (measured at a 1 km^2^ scale) as a function of average ecoprovince road density across boreal Canada during the summer (top) and winter (bottom) as modeled using a generalized functional response approach (GFR)

The seasonal boreal Canada‐wide GFR models (Figure 3) showed that wolves selected habitat patches (i.e., 1 km^2^ areas) with higher NDVI values (Table 2). The selection of patches with higher road density varied with average ecoprovince road density, supporting a functional response to roads (see positive interaction coefficient in Table 2). Specifically, in the summer, wolves selected less the patches with high road density in ecoprovinces with relatively low road densities (i.e., <0.075 km/km^2^; Figure 4, top), but selected patches with higher road density in ecoprovinces with relatively high average road densities (i.e., >0.075 km/km^2^). Similar selection patterns were observed in winter, with wolves showing a gradual shift toward selection of habitat patches of high road densities, as average ecoprovince‐scale road densities increased (Figure 4, bottom).

**Table 2:** Model coefficients (*β*) standards errors (*SE*), *z*‐values and *p*‐values for covariates of boreal Canada‐wide scale (i.e., generalized functional response, GFR) wolf resource selection functions in the summer and winter

	Summer				Winter			
	*β*	*SE*	*z*‐value	*p*‐value	*β*	*SE*	*z*‐value	*p*‐value
NDVI	0.18	0.09	2.01	.04	0.43	0.06	7.44	<.01
Cutblock density	−8.15	0.83	−9.87	<.01	−4.02	0.38	−10.68	<.01
Road density	−1.88	0.22	−8.46	<.01	−0.68	0.13	−5.29	<.01
Ecoprov road density (rds_E)^a^	0.80	0.66	1.22	.22	0.00	0.28	0.00	1.00
Ecoprov cutblock density (cut_E)^a^	−1.90	1.01	−1.89	.06	2.83	0.46	6.13	<.01
Cutblock density * cut_E	5.86	2.11	2.78	.01	8.26	1.30	6.37	<.01
Road density * rds_E	5.33	0.65	8.15	<.01	1.23	0.37	3.36	<.01
Road density * cut_E	−8.24	1.06	−7.81	<.01	1.09	0.62	1.75	.08
Cutblock density * rds_E	−8.23	1.36	−6.04	<.01	−1.34	0.91	−1.47	.14

a Ecoprov covariates (rds_E and cut_E) are the average road density and cutblock density values sampled in each ecoprovince (sampled from locations available to wolves).

**Figure 5:**
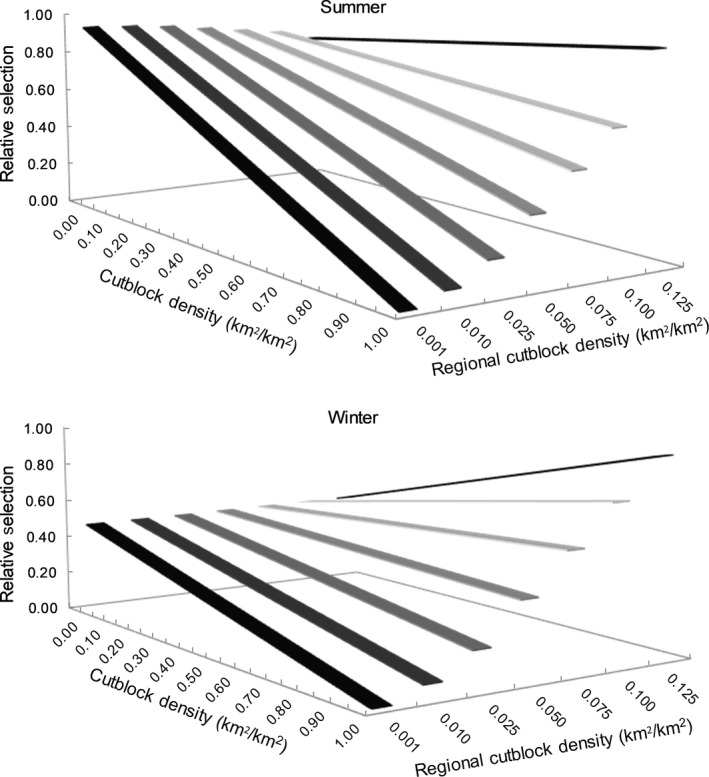
Relative selection by wolves of cutblock density (measured at a 1 km^2^ scale) as a function of average ecoprovince cutblock density across boreal Canada during the summer (top) and winter (bottom) as modeled using a generalized functional response approach (GFR)

Similarly, the selection of patches with higher cutblock density varied with average ecoprovince cutblock density, supporting a functional response to forestry cutblocks (see positive interaction coefficient in Table 2). In the summer, wolf selection of patches with higher cutblock density became pronounced as average ecoprovince cutblock density increased, and wolves selected patches with slightly higher cutblock density in the ecoprovince with the highest cutblock density (i.e., 0.125 km^2^/km^2^; Figure 5, top). In winter, wolves selected less the patches with high cutblock density in ecoprovinces with lower average cutblock densities, but were more likely to select patches with high cutblock density in ecoprovinces with higher average cutblock densities (i.e., >0.075 km^2^/km^2^; Figure 5, bottom).

### 3.2 Trade‐offs in selection of forestry cutblocks and roads

**Figure 6:**
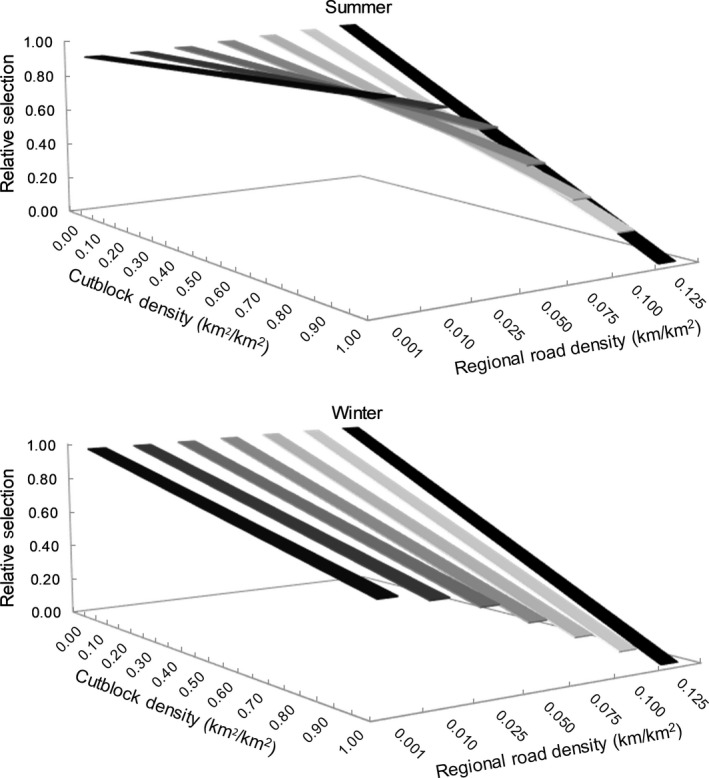
Relative selection by wolves of cutblock density (measured at a 1 km^2^ scale) as a function of average ecoprovince road density across boreal Canada during the summer (top) and winter (bottom) as modeled using a generalized functional response approach (GFR)

**Figure 7:**
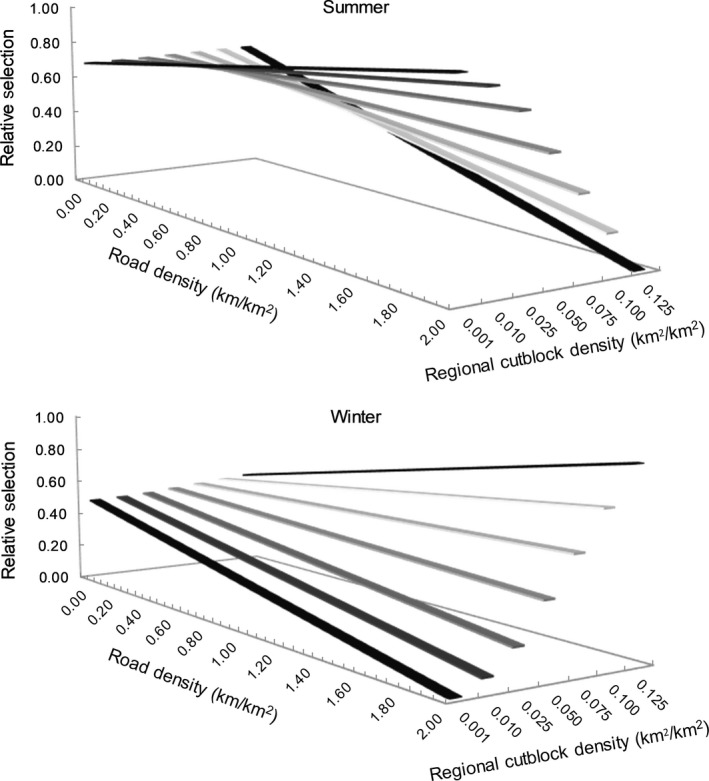
Relative selection by wolves of road density (measured at a 1 km^2^ scale) as a function of average ecoprovince cutblock density across boreal Canada during the summer (top) and winter (bottom) as modeled using a generalized functional response approach (GFR)

We found a significant negative interaction between habitat patch cutblock density and ecoprovince‐scale road density in wolf resource selection (Table 2), indicating a functional response with trade‐offs. In the summer, wolves selected patches with higher cutblock density in ecoprovinces with lower average road densities (i.e., <0.025 km/km^2^; Figure 6, top), but selected less the patches with higher cutblock density in ecoprovinces with higher average road densities. In winter, wolves generally selected less the patches with higher cutblock density in ecoprovinces with higher average road densities (Figure 6, bottom). Similarly, we found a significant negative interaction between patch‐scale road density and average ecoprovince cutblock density in wolf resource selection (Table 2), also indicating a functional response with trade‐offs. In summer, wolves selected patches with higher road density in ecoprovinces with lower average cutblock densities (i.e., <0.025 km^2^/km^2^; Figure 7, top), but selected less the patches with higher road density in ecoprovinces with higher average cutblock densities. In winter, wolves selected less the patches with higher road density in ecoprovinces with lower average cutblock densities (i.e., <0.100 km^2^/km^2^; Figure 7, top) but selected patches with high road density in ecoprovinces with higher average cutblock densities (Figure 7, top).

### 3.3 Model generalizability and prediction of wolf use

Our winter and summer habitat selection models had very good to good generalizability in predicting wolf distribution. Very good generalizability was found in predictions of wolf use in the western taiga shield and eastern boreal plain ecoprovinces in the winter or summer, in the boreal foothills and western boreal shield ecoprovinces in the winter, and in the midboreal shield in the summer (*R* ^2^ ≥ .99, −6 ≤ Intercept ≤ 1, 0.96 ≤ Slope ≤ 1.12, *p* < .01; Table 3). Model generalizability was still good in the other ecoprovinces including 4 for the winter and 3 for the summer (*R* ^2^ ≥ .80, −20 ≤ Intercept ≤ 11, 0.87 ≤ Slope ≤ 1.22 *p* < .01; Table 3).

**Table 3:** K‐fold cross validation statistics, where the GFR model was fit iteratively on subsets of data with just one ecoprovince withheld (subsets used to construct the model), and we then compared predicted and observed distributions of wolf locations for the ecoprovince withheld

Season	Ecoprovince	Slope	*p*‐value	Intercept	*R* ^2^
Summer	Western taiga shield	1.02	<.01	−1	1.00
	Central boreal plains	0.94	<.01	10	.96
	Western boreal shield	0.87	<.01	11	.96
	Boreal foothills	1.07	<.01	−9	.89
	Eastern boreal plains	1.06	<.01	−1	1.00
	Eastern boreal shield	1.22	<.01	−20	.96
	Midboreal shield	0.96	<.01	1	.99
Winter	Western taiga shield	1.03	<.01	−1	.99
	Central boreal plains	1.08	<.01	−20	.80
	Western boreal shield	1.02	<.01	−4	1.00
	Boreal foothills	1.09	<.01	−1	1.00
	Eastern boreal plains	1.12	<.01	−6	.99
	Eastern boreal shield	0.95	<.01	8	.97
	Midboreal shield	1.10	<.01	−7	.99

## 4 DISCUSSION

This study describes the response of a large predator to variation in human‐caused habitat alterations across Canada's boreal forest, a vast landscape with a high diversity of human footprint conditions. Wolves' selection of roads and forestry cutblocks varied by season and across ecoregions. Our results confirm that while wolves are habitat generalists, they adapt and specialize their resource use to specific environments depending on resource availability (Paquet et al., 2010). Our results are novel in that they also highlight how functional responses can effectively capture the flexibility of animal selection and meet the challenges in predicting the effects of humans on wildlife. Functional responses are not new in the wolf literature as they were amply described for prey selection (see Dale, Adams, & Bowyer, 1994; Zimmermann, Sand, Wabakken, Liberg, & Andreassen, 2015). Our results indicate the species' aptitude for functional responses in habitat selection too, where similar mechanisms of selection and “switching” may play a role.

Both in summer and winter, wolves selected higher road density habitat patches in ecoprovinces with higher road densities (i.e., the “road resource” become more desirable despite it being more available), and they selected higher cutblock density habitat patches in ecoprovinces with higher cutblock densities (i.e., the “cutblock resource” become more desirable despite it being more available). These results suggested wolves dynamically select both types of human footprint, which may facilitate wolf predation as road may be used to travel efficiently and encounter prey in cutblock areas. Cutblocks provide early seral forest habitat that provides food for wolf prey (Gagné et al., 2016), and thus may support higher prey densities (Bowman et al., 2010; Peters et al., 2012; Rempel, Elkie, Rodgers, & Gluck, 1997). We also found that wolves selected patches of higher vegetation productivity (i.e., high NDVI values), which likely supported higher prey densities (Street et al., 2015). Roads may increase the travel efficiency of wolves (Dickie et al., 2017; Latham et al., 2011; Whittington et al., 2005) and even facilitate wolf predation on prey (Paquet et al., 2010; Whittington et al., 2011). However, the benefits of roads and cutblocks for wolves may not be realized at low densities of these footprint types, where they may be perceived by wolves as unusual landscape features.

We also found a novel and significant functional response in how wolves traded off between human footprint types (see negative interaction coefficients in Table 2, Figures 6 and 7). At low densities of roads and cutblocks (i.e., little benefits provided, as explained above), wolves may select even less each footprint type (roads or cutblocks), if human activities associated with roads or cutblocks increase the probability of wolf mortality through human‐wolf interactions (Benson et al., 2015; Lovari et al., 2007). Alternatively, it may be that in landscapes with high densities of each footprint type, the risk of human interaction is equally high across habitat patches, and thus avoidance of these features is no longer a beneficial strategy for reducing mortality risk. Where high densities of only one type of human footprint occur, wolves may select habitat patches with higher densities of that footprint because these features may facilitate predation (by facilitating movement or providing access to higher prey density).

Our results revealed that wolf selection of the two footprint types did not increase with availability simultaneously. Thus, human activity may limit wolf use of habitat, as wolves may only be able to maximize their use of cutblocks in regions where road densities are low, for example. By comparison, high road densities may reduce wolf habitat suitability despite the potential for the landscapes to support higher prey densities (Fisher & Wilkinson, 2005; Gagné et al., 2016). Whereas wolves may be able to trade‐off between different human footprint types, ultimately, wolves may not select habitat patches with high densities of both types of human footprint because wolves may not tolerate cumulative effects of multiple human activities.

Overall, human footprint and prey density, and its accessibility through using roads especially in the winter likely determined wolf habitat selection. Wolves may optimize the use of roads to locate prey in landscapes with low prey densities, and switch to using cutblocks in landscapes with higher prey densities (Kittle et al., 2017). However, we observed that in winter wolves selected habitat patches with high road density even in ecoprovinces with high cutblock density. Newton et al. (2017) found compensatory selection for roads over natural linear features, presumably because roads were more energetically favorable for wolf travel (i.e., lower sinking depths). Moreover, mortality risks on roads may be reduced in winter because human activity related to forestry is typically less than in summer in remote regions, such as our study area (Houle et al., 2010).

Our GFR models could predict where wolves were most likely to occur, given habitat features assessed at fine scale (i.e., at locations used by wolves) and at assessed at broad scale (i.e., at locations available to wolves within ecoprovinces), and given their interaction. The strength of GFRs is their ability to test for generalizable effects of habitat availability on species distribution, and more accurately predict those effects across a wide range of habitats. We found our GFR model to be generalizable, as it predicted actual use by wolves in each ecoprovince, with a few minor exceptions.

We caution when using this model to predict wolf use in the central boreal plains ecoprovince year‐round, the boreal foothills and eastern boreal shield during the summer, and the midboreal shield and eastern boreal plains during the winter. The model appeared to underpredict the use of suboptimal habitat and overpredict the use of optimal habitat for wolves in these ecoprovinces. It is difficult to distinguish whether this issue was caused by model overfitting or simply a lack of transportability of the model to these ecoprovinces, due to unique ecological conditions. Model fit may also depend on the prey communities present in each ecoprovince. For example, beaver, which are less associated with cutblocks (i.e., a variable that is prominent in our model), are important prey in the central boreal plains (Latham et al., 2013), whereas moose, which are strongly associated with cutblocks, may be more important in other areas. Finally, environmental data sets comprehensively covering the telemetry period and the whole study area were not available. In addition, we had to rely on datasets that were diligently assembled, but did not account for the temporally dynamic nature of changes in forest landscapes. For these reasons, we recommend that for this, or any other broad‐scale species distribution prediction, the model be tested with independent data collected at regular time intervals in the area where it will be applied, prior to using it to make management decisions.

## 5 CONSERVATION IMPLICATIONS

Habitat selection models like the one developed in this study are a useful tool to show or predict how human‐induced changes to habitat influence the ecology of wildlife species, and potentially the interactions of species. Human modifications of landscapes are typically complex, which can make predicting and managing human effects on wildlife and ecosystems a significant challenge. However, some patterns are predictable. The road and forestry cutblock footprints accounted for in this study have vastly different, but predictable environmental impacts, as illustrated here by the different and interacting effects that roads and cutblocks had on wolves. In addition, the effects of humans on a given species likely have indirect effects on other wildlife species, further complicating our ability to manage human influence on ecosystems. For example, in our study area a key concern is the indirect effect of humans on woodland caribou mediated by wolves through apparent competition (DeCesare, Hebblewhite, Robinson, & Musiani, 2010; Fortin et al., 2017; Holt, 1977). Woodland caribou are highly sensitive to predation, and our results confirm that human footprint in caribou range could enable increased distribution of wolves, potentially resulting in higher predation rates on this threatened species (Wittmer et al., 2005). Overall, our findings demonstrate direct effects of human‐caused habitat alterations on wolves, and potentially support indirect effects rippling on prey and vegetation. Therefore, this work could serve to help understand, predict and manage human impacts toward conservation objectives.

Our model is generalizable to all ecoregions encompassing the vast boreal forest zone of Canada. Indeed, the interactions between different types of human footprint at a regional scale were integral to understanding the nuances of human footprint effects on wolves. Similar methodological approaches could be used for predicting wolf habitat use in other boreal forests, for example of Alaska or Eurasia, or across similarly large geographic areas, or into the future, as road and forestry developments or other developments increase over time –that is, an analysis that we could not accomplish due to lack of longitudinal data on human development. In future studies, we recommend that scientists and conservation managers consider the contextual and interacting effects of human footprints when assessing the impacts of human development on wildlife.

## CONFLICT OF INTEREST

None declared.

## AUTHORS CONTRIBUTIONS

EWN, DF, ADML, MCL, EM, BRP, FS provided wolf data included in this analysis. The research approach was designed and developed by all authors in collaboration (TBM, CAJ, MH, EWN, DF, JMF, ADML, MCL, PDM, EM, PCP, BRP, FS, FS, and MM). TBM lead the statistical analyses. TBM, MM, CAJ, and BRP lead the write‐up components of this work.

## Data Availability

All Resource Selection Function model outputs and maps, and all environmental GIS layers and maps are available through the repository: https://doi.org/10.5061/dryad.q9j281m
