# Supplementary material for: Functional response of wolves to human development across boreal North America
Source: Ecol Evol. 2019 Aug 30;9(18):10801–15. doi: 10.1002/ece3.5600 (PMC6787805; doi:10.1002/ece3.5600)

# Supporting information

## Table S1. Wolf GPS data contributors, Canadian Province or Territory where the studies were conducted, and animal care certifications and protocols.

| Contributor. | Province or Territory | Animal care certification | Protocol or Permit No. |
| --- | --- | --- | --- |
| B.C. Boreal Caribou Research and Effectiveness Monitoring Board | British Columbia | B.C. Ministry of Forest, Lands and Natural Resource Operations Animal Care Committee | FJ14-156487 |
| B.R. Patterson and J.M Fryxell | Ontario | University of Guelph’s Animal Utilization Protocol and the Ontario Ministry of Natural Resources Wildlife Animal Care Committee | 10/11/12-218 |
| E. Merrill, N. Webb and P. Knamiller | Alberta | University of Alberta Animal Care and Use Protocol | 391305, 353112, 411601 and 411601 |
| S. Boutin and H. Bohm | Alberta | University of Alberta Animal Care | ACUC Study Id. AUP00000040 |
| F. Schmiegelow | Alberta | University of Alberta Animal Care | 96-99D and 2003-32D |
| Manitoba Hydro (F. Scurrah) | Manitoba | The Province of Manitoba Sustainable Development, Wildlife Branch |  |
| M. Arienti and D. Latham | Alberta | University of Alberta Biosciences Animal Policy and Welfare Committee | 471503 |
| M. Arienti and D. Latham | Alberta | Alberta Environment and Resource Development Wildlife Research and Collection Permit | 23428 and 23669 |
| D. Fortin | Quebec | Comité de protection des animaux of the Université Laval | CPAUL: 2008026-3; 2015015-1 |
| P. McLoughlin | Saskatchewan | University of Saskatchewan 2014 | 20130127 |

## Figure S1. Boreal forest of Canada and its seven ecoprovinces, locations used by 172 GPS collared wolves, and locations considered as “available” to these individual wolves.


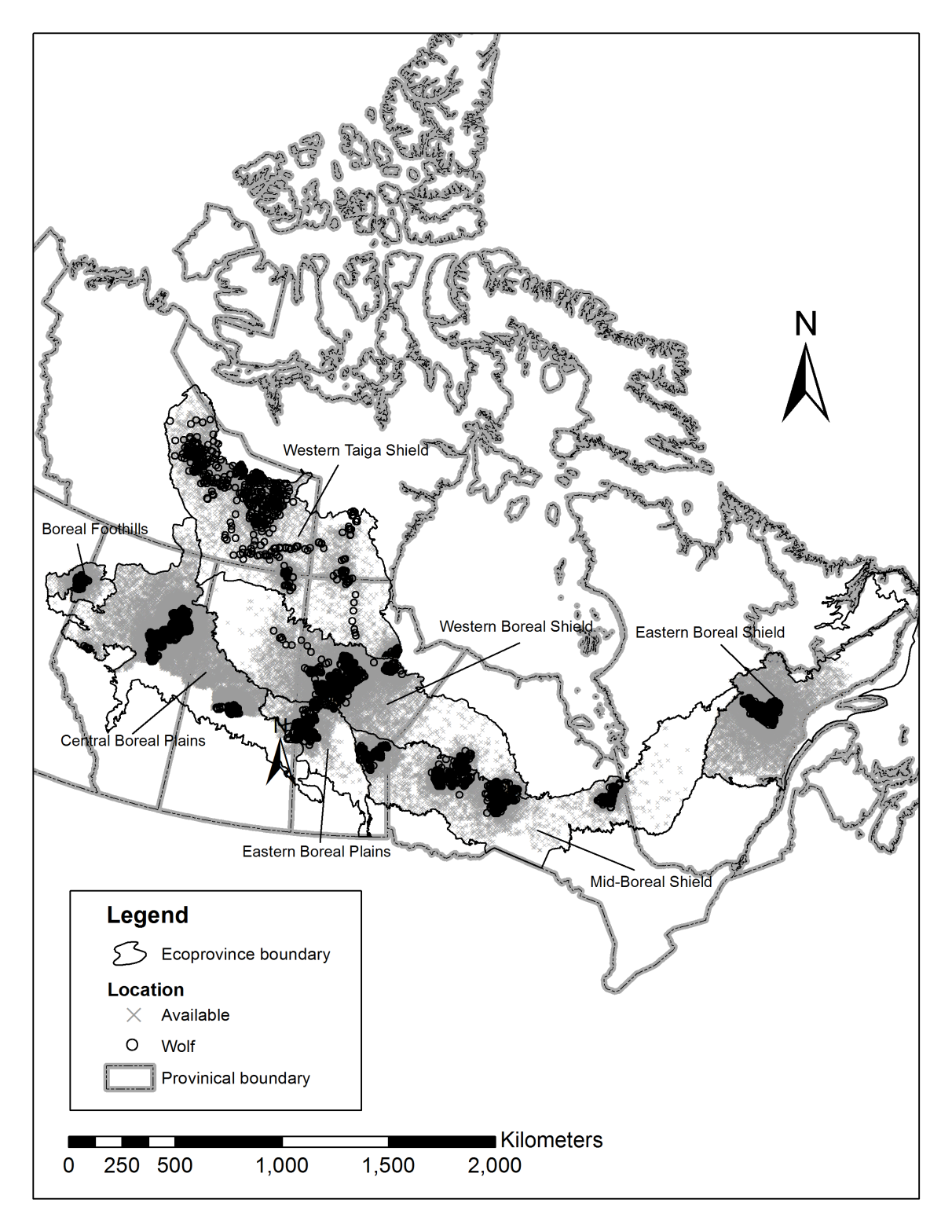

Supplement: Supplementary file 1 [file ECE3-9-10801-s001.docx]
